# Costs and effects of screening and treating low risk women with a singleton pregnancy for asymptomatic bacteriuria, the ASB study

**DOI:** 10.1186/1471-2393-12-52

**Published:** 2012-06-21

**Authors:** Brenda M Kazemier, Caroline Schneeberger, Esteriek De Miranda, Aleid Van Wassenaer, Patrick M Bossuyt, Tatjana E Vogelvang, Frans JL Reijnders, Friso MC Delemarre, Corine JM Verhoeven, Martijn A Oudijk, Jeanine A Van Der Ven, Petra N Kuiper, Nicolette Feiertag, Alewijn Ott, Christianne JM De Groot, Ben Willem J Mol, Suzanne E Geerlings

**Affiliations:** 1 Department of Obstetrics and Gynaecology, Academic Medical Centre, Amsterdam, the Netherlands; 2 Department of Infectious Diseases, Centre for Infection and Immunity Amsterdam (CINIMA), Academic Medical Centre, Amsterdam, the Netherlands; 3 Department of Neonatology, Emma Children’s Hospital Academic Medical Centre, Amsterdam, the Netherlands; 4 Department of Clinical Epidemiology, University of Amsterdam, Amsterdam, the Netherlands; 5 Department of Obstetrics and Gynaecology, Diakonessenhuis Utrecht, Utrecht, the Netherlands; 6 Department of Obstetrics and Gynaecology, Slingeland Hospital, Doetinchem, the Netherlands; 7 Department of Obstetrics and Gynaecology, Elkerliek Hospital, Helmond, the Netherlands; 8 Department of Obstetrics and Gynaecology, Máxima Medical Centre, Veldhoven, the Netherlands; 9 Department of Obstetrics and Gynaecology, University Medical Centre Utrecht, Utrecht, the Netherlands; 10 Ultrasound centre Espérance, Arnhem, the Netherlands; 11 Ultrasound centre FARA, Ede, the Netherlands; 12 Ultrasound centre VCO echopunt, Amsterdam, the Netherlands; 13 Laboratory for infectious diseases, Groningen, the Netherlands; 14 Department of Obstetrics and Gynaecology, VU Medical Centre, Amsterdam, the Netherlands

## Abstract

**Background:**

The prevalence of asymptomatic bacteriuria (ASB) in pregnancy is 2-10% and is associated with both maternal and neonatal adverse outcomes as pyelonephritis and preterm delivery. Antibiotic treatment is reported to decrease these adverse outcomes although the existing evidence is of poor quality.

**Methods/Design:**

We plan a combined screen and treat study in women with a singleton pregnancy. We will screen women between 16 and 22 weeks of gestation for ASB using the urine dipslide technique. The dipslide is considered positive when colony concentration ≥10^5^ colony forming units (CFU)/mL of a single microorganism or two different colonies but one ≥10^5^ CFU/mL is found, or when Group B Streptococcus bacteriuria is found in any colony concentration. Women with a positive dipslide will be randomly allocated to receive nitrofurantoin or placebo 100 mg twice a day for 5 consecutive days (double blind). Primary outcomes of this trial are maternal pyelonephritis and/or preterm delivery before 34 weeks. Secondary outcomes are neonatal and maternal morbidity, neonatal weight, time to delivery, preterm delivery rate before 32 and 37 weeks, days of admission in neonatal intensive care unit, maternal admission days and costs.

**Discussion:**

This trial will provide evidence for the benefit and cost-effectiveness of dipslide screening for ASB among low risk women at 16–22 weeks of pregnancy and subsequent nitrofurantoin treatment.

**Trial registration:**

Dutch trial registry: NTR-3068

## Background

Asymptomatic bacteriuria (ASB) is the presence of significant bacteriuria without the symptoms of a urinary tract infection (UTI). ASB occurs in 2-10% of pregnant women [1]. ASB during pregnancy can lead to serious complications for both mother and child. The incidence of ASB is similar in both pregnant and non-pregnant women [2]. Pregnant women with ASB, however, develop pyelonephritis more often, probably due to the anatomic and physiologic changes that occur during pregnancy, which may facilitate bacterial growth and ascending of bacteria to the kidneys [3]. If left untreated, 20% to 40% of pregnant women with ASB will develop pyelonephritis [2,4,5].

Other possible adverse effects, such as preterm delivery and delivering a low birth weight infant are less well established. Preterm delivery is the main cause of neonatal mortality and morbidity worldwide. The causal mechanisms remain unknown. One of the hypotheses is that endotoxins released by bacteria cause uterine contractions leading to preterm delivery.

### Necessity of screening for ASB

Some national guidelines [1,6,7] recommend ASB screening and treatment in pregnancy. However, these guidelines are based on research conducted more than 30 year ago. Furthermore, our knowledge about methodology and the causing mechanisms of pyelonephritis has developed. Since the methods used in these early studies are inadequately described, interpretation of this evidence is difficult and the conclusions that can be drawn are limited.

Although many articles have been published on ASB in pregnancy, the role of ASB in perinatal outcomes is not clear [8]. Another problem is that most recent papers focus on the best treatment strategies instead of evaluating the actual need for a screen and treat program.

The widespread use of antibiotics as a consequence of the screening programs is reason for concern. The ORACLE Children Study II [9] showed increased functional impairment in children from mothers using antibiotics for the prevention of preterm labour in pregnancy. Other studies also showed an adverse effect of antibiotics on the offspring, such as increased antibiotic resistance in late-onset serious bacterial infections [10]^;^[11]. Considering these results, one should carefully balance the consequences of bacteriuria in pregnancy against the possible effects of antibiotics, before routinely treating all women with ASB.

### Antibiotics choice and duration

There is no consensus in the literature on either the duration of therapy or the choice of antibiotic. As a result practice is guided by national or local practices and resistance patterns[8]. A recent Cochrane review on the treatment duration for ASB underlines this lack of clear evidence on the best treatment[12].

The latest guidelines of the Infectious Disease Society of America (IDSA), published in 2005, recommend for the treatment of ASB a 3 to 7-day course for the treatment of ASB that includes sulphonamides, nitrofurantoin, nalidíxic acid, amoxicillin or trimethoprim [1].

E *coli* is the most common pathogen found in ASB [3] and treatment should be targeted to the most common pathogens. Nitrofurantoin has proven to be safe in pregnancy [13,14] with very low resistance levels in Netherlands [15]. Nitrofurantoin is first choice in the treatment of cystitis in pregnancy in the Netherlands [16,17].

The Dutch guidelines of the Dutch Society of Obstetrics and Gynaecology (NVOG ) and the Dutch General Practitioners Society (NHG) do not currently recommend routine screening and treatment of ASB in pregnancy[16,17] because convincing evidence is lacking. The Netherlands is one of the few countries which can still properly investigate this important question because a non treating policy of woman with ASB does not violate the guideline.

In view of the lack of good clinical evidence on the subject and the resulting practice variation, we think that an appropriately designed clinical trial evaluating the costs and effects of a screen and treat program is urgently needed. In a national cohort study women will be screened for ASB with the dipslide technique. Women with ASB will be randomised to either placebo or nitrofurantoin.

## Methods/design

### Outline

The study will be a prospective cohort screening study with a randomised clinical trial embedded. We will screen a large cohort of women with low risk singleton pregnancies at 16–22 weeks gestation with the dipslide technique. Women with a positive dipslide without symptoms of a UTI will be randomly allocated to receive either nitrofurantoin or placebo for 5 days. To mask women for their bacteriuria status a small sample of women without ASB will also be offered the possibility to participate in the study. Women without ASB will always receive placebo. Both women and researchers will be unaware of the bacteriuria status and treatment allocation. Because of the blinding of bacteriuria status, women with GBS bacteriuria will not receive intrapartum antibiotics in the absence of other risk factors.

The objective of the randomized trial is to evaluate whether nitrofurantoin treatment of women with ASB is effective in reducing the risk of preterm delivery and/or pyelonephritis (primary outcome) and adverse neonatal outcome (secondary outcome).

### Participants/eligibility criteria

The study is set in the Dutch Obstetric Consortium, a collaboration of obstetric practices in the Netherlands. A variety of clinics, including university hospitals, teaching hospitals, non-teaching hospitals, ultrasound centres and midwifery practices will participate in this trial. Women with a singleton pregnancy without symptoms of a urinary tract infection at 16–22 weeks of gestation can participate in the *ASB screening* study.

Women with a history of spontaneous preterm delivery before 34 weeks, signs of threatening preterm delivery, foetal congenital malformations, use of antibiotics at time of screening, known G6PD deficiency or allergy to nitrofurantoin or risk factors for complicated UTI (diabetes mellitus, immunosuppressive medication, functional or structural abnormalities of the urinary tract) are excluded from the screening study.

### Procedures, recruitment, randomisation and collection of baseline data

#### ASB screening trial

In the ASB *screening* trial, we will offer low risk women with a singleton pregnancy the possibility to be screened for ASB with the dipslide technique. At the 16^th^ week of gestation the timing of screening is considered optimal [4,18]. For logistic reasons we decided to do the screening at the same time the structural ultrasound scan for foetal abnormalities is performed in the Netherlands.

A single dipslide (Uricult®, Orion Diagnostica) consisting of two different media (green cysteine lactose electrolyte deficient medium and reddish MacConkey medium) will be used to diagnose ASB. Previous research showed that the dipslide is a promising alternative for the conventional culture, which is currently considered the gold standard[19,20]^;^[3]. The dipslide has 98.0% sensitivity and 99.6% specificity for detecting ASB in pregnancy [21]. Urinary culture is not feasible in the Dutch antenatal care system since 70% of Dutch women attend antenatal care at a midwifery practice. Hence, there is no direct access to a microbiology laboratory to perform the cultures.

The dipslide will be inoculated with midstream urine at a hospital, ultrasound centre or midwifery practice. Perineal cleansing prior to voiding is not necessary since it does not decrease bacterial contamination [22]. The dipslides will be sent by mail to the laboratory for infectious diseases in Groningen, the Netherlands the same day. Laboratory technicians will read the dipslide directly when incubated for 2 to 3 days at room temperature. If no colonies have been formed, the dipslide will be incubated for another 24 hours at 35^o^ Celsius.

Dipslides are considered positive when the colony concentration is ≥ 10^5^ CFU/mL of a single microorganism or when two different colonies are present but one has a concentration of ≥10^5^ CFU/mL. When Group B Streptococcus (GBS) is found also colony concentrations <10^5^ CFU/mL are considered positive because treatment may still be beneficial [23]. When more species are present the dipslide is considered contaminated.

All women participating in the ASB *screening* study will receive two questionnaires. The first questionnaire contains questions about ethnicity, marital status, length, weight, education, smoking, alcohol- and drug use, co morbidity, parity, inter-pregnancy interval and exclusion criteria. This questionnaire will be filled out at the moment of screening. The second questionnaire will be sent to the participants 6 weeks after their due date. It contains questions about UTIs, use of antibiotics and hospital admissions during this pregnancy, pregnancy complications and pregnancy outcomes. Furthermore we will ask all women in the screening study for informed consent to collect data on their pregnancy outcomes.

#### ASB treatment trial

Women eligible for the ASB *treatment trial* will be identified by the laboratory personnel participating in this study. A research midwife or -nurse will contact these women for participation in the treatment trial.

Before entering the study, women will be informed about the aims, methods, reasonably anticipated benefits and potential hazards of the study. Participation is voluntary and withdrawal of consent to participate is possible at any time during the study. After giving sufficient information, written informed consent will be asked for. Women eligible for the ASB *treatment trial* who do not give informed consent, will be registered. Nitrofurantoin will not be offered to these women.

After participant data have been entered in a web based database, computerized randomisation will take place. The women with ASB will be randomised 1:1 for nitrofurantoin and placebo. Women without ASB (used for blinding of bacteriuria status) will always receive placebo.

### Intervention

Each study participant will be given a jar labelled “ASB *treat* study” which contain either 100 mg capsules of nitrofurantoin (Nitrofurantoin MC, TioFarma, the Netherlands) or identical-appearing capsules of placebo (TioFarma, the Netherlands).

The oral study medication will be self-administered twice a day for 5 consecutive days. The label codes indicating nitrofurantoin or placebo are blinded for the participants and researchers. The deblinding list is present in the central pharmacy. For emergency cases, a closed envelope with the label codes is also available at the study centre. The data will be disclosed to the researchers in case of emergencies and otherwise after collection and analysis of the primary outcomes. Researchers involved in the follow up program of the offspring of women participating in this study will remain blinded for a longer period. For purpose of the interim analysis the label codes will become available to the epidemiologist involved in the study as A and B.

All participants who receive study medication -i.e. the screen positives as well as the random subsample of screen negatives-, will have follow up dipslides done 1 week after the end of treatment. Participants with a persistent positive culture and a subsample of participants with a negative culture will receive again (blinded) study medication. The participants who received nitrofurantoin will again receive nitrofurantoin, placebo participants will again receive placebo. One week after this second intervention, once again a dipslide is performed.

### Outcome measures

The primary outcomes for the screen and treat study are the development of pyelonephritis and delivery before 34 weeks. Pyelonephritis is defined as an episode of fever (≥38.0°C), symptoms (nausea, vomiting, chills, costo-vertebral tenderness) and a positive urine culture. The primary outcome measure will be recorded 6 weeks after the expected due date.

Secondary outcome is an adverse neonatal outcome (death or severe morbidity). The composite morbidity rate contains the following variables: severe respiratory distress syndrome (RDS), bronchopulmonary dysplasia (BPD), periventricular leucomalacia > grade 1, intracerebral haemorrhage > grade II, necrotizing enterocolitis (NEC) > stage 1, proven sepsis (including GBS sepsis) and death before discharge from the nursery.

Other outcome parameters are: neonatal weight, time to delivery, preterm birth rate before 32 and 37 weeks, days of admission in neonatal intensive care unit, maternal morbidity (including UTI), chorioamnionitis, maternal admission days for (threatened) preterm labour and/or pyelonephritis and costs.

Furthermore, we will look at growth, physical condition and neurodevelopmental outcome of the child at 24 months (corrected) age.

Apart from clinical outcome, the cost-effectiveness of screening for ASB (as done in ASB *screening*), and subsequent treatment in cases of ASB (as done in ASB *treatment trial*), will be assessed.

### Follow up of women and infants

We plan follow-up of infants at the corrected age of 24 months with the Ages & Stages Questionnaires (ASQ) and the Child Behavioural Checklist (CBCL). The checklists will be sent to the parents of the child. In case the parents do not return the questionnaire, a reminder will be sent. We will also ask them to report length, weight medical history and medical consumption of the child.

### Data analysis

The results of the screen cohort will allow us to describe the incidence of ASB in the Netherlands as well as to explore risk factors for developing ASB or pyelonephritis.

The results of the randomised clinical trial will be analyzed according to the intention to treat principle. The effectiveness of nitrofurantoin versus placebo will be assessed by calculating relative risks and 95% confidence intervals. The number of primary- and secondary outcomes will be compared between the ASB positive and ASB negative (ASB screen study) and treatment and control (ASB treat study) groups.

#### Interim analysis

Interim analysis will be monitored by an independent Data Safety Monitoring Committee. We plan an interim analysis for futility and safety after 100 participants in the ASB *treat* study. This analysis will be done by an independent person who will be unaware of the allocation of treatment when data are judged for effectiveness.

### Statistical issues

#### Sample size calculation

Among women positive for ASB, we anticipate the occurrence of the primary outcome (delivery before 34 weeks and/or pyelonephritis) to be 10% in the treatment group and 25% in the no treatment group. If ASB is not treated, 20% of pregnancies with ASB will be complicated by pyelonephritis compared to 2% of pregnancies without ASB [24]. Treatment of ASB results in a decrease of pyelonephritis compared to women who were not treated. Using a two-sided test with an alpha 0.05 and a beta of 0.8, 220 women with ASB (110 per arm) are needed in the study. Anticipating a 5% incidence of ASB, we need to screen 4.400 women. Obviously, final recruitment statistics will depend on the screen positive rate, which is one of the study questions. From our previous experience in the Triple P trial [25] we learned that Dutch women are very reserved in taking study medication for asymptomatic conditions in pregnancy. If during this trial it becomes clear that very few women consent to participate in the ASB treat study we will not increase our screening cohort indefinitely to reach our planned randomisations.

### Economic evaluation

#### General considerations

The economic analysis will be performed from a societal perspective. Both costs and outcomes will be discounted with a discount rate of 5%. The economic analysis of the trial itself is not of interest. If nitrofurantoin is found to decrease the probability of pyelonephritis or preterm delivery, then the savings due to decreased maternal and neonatal admission will always outweigh the costs of nitrofurantoin, which are negligible. The true economic question to be answered, when the treatment trial shows a beneficial effect, is whether the costs of screening (number needed to screen to detect one woman with ASB) outweigh the cost reduction and health benefits from treatment with nitrofurantoin.

#### Cost analysis

The study design will enable us to compare the costs and effects of the following strategies:

I. no screening for ASB

II. screening for ASB and treatment of women with ASB

For each of these strategies, we will calculate the costs as well as the effects in terms of pyelonephritis or preterm delivery. In the cost-effectiveness analysis, we will then calculate the costs per prevented case of pyelonephritis or preterm delivery. Thus, the cost-effectiveness analysis will assess the balance between number needed to treat and number needed to screen.

### Data safety monitoring committee

Serious Adverse Events (SAEs) and Suspected Unexpected Serious Adverse Reactions (SUSARs) will be reported to a Data Safety Monitoring Committee (DSMC). The DSMC can order to perform an extra interim analysis and, if indicated, terminate the trial prematurely.

### Ethical considerations

This study is approved by the National Central Committee on Research involving Human Subjects (CCMO - NL35375.018.11) and by the ethics committee of the Academic medical centre Amsterdam (ref. no MEC 2011_073).

## Discussion

To our knowledge there are no other ongoing trials in the Netherlands or other countries, evaluating a screen and treat strategy for ASB with the dipslide technique (http://www.controlled-trials.com/mrCT/). This trial will provide evidence for the usefulness and cost-effectiveness of screening for ASB at 16–22 weeks of pregnancy with a dipslide and subsequent nitrofurantoin treatment among low risk women.

## Abbreviation

ASB, Asymptomatic bacteriuria; CFU, Colony forming units; ml, Millilitre; UTI, Urinary tract infection; SAE’s, Serious adverse Events; SUSARS, Supspected Unexpected Serious Adverse Reactions; DSMC, Data Safety Monitoring Committee.

## Competing interests

The authors declare that they have no competing interests.

## Authors’ contributions

SEG, CJG and BWM were involved in conception and design of the study. BMK, CS and SEG, drafted the manuscript. All authors mentioned in the manuscript are members of the ASB study group. They participated in the design of the study during several meetings and are local investigators in the participating centres. All authors edited the manuscript and read and approved the final manuscript.

## Pre-publication history

The pre-publication history for this paper can be accessed here:

http://www.biomedcentral.com/1471-2393/12/52/prepub

## References

[B1] Nicolle LE Bradley S Colgan R Rice JC Schaeffer A Hooton TM Infectious Diseases Society of America guidelines for the diagnosis and treatment of asymptomatic bacteriuria in adults Clin Infect Dis 2005 40 643 654 10.1086/427507 15714408

[B2] Patterson TF Andriole VT Detection, significance, and therapy of bacteriuria in pregnancy. Update in the managed health care era Infect Dis Clin North Am 1997 11 593 608 10.1016/S0891-5520(05)70375-5 9378925

[B3] Macejko AM Schaeffer AJ Asymptomatic bacteriuria and symptomatic urinary tract infections during pregnancy Urol Clin North Am 2007 34 35 42 10.1016/j.ucl.2006.10.010 17145359

[B4] Millar LK Cox SM Urinary tract infections complicating pregnancy Infect Dis Clin North Am 1997 11 13 26 10.1016/S0891-5520(05)70339-1 9067782

[B5] Kass EH Bacteriuria and pyelonephritis of pregnancy Arch Intern Med 1960 105 194 198 10.1001/archinte.1960.00270140016003 14404662

[B6] Lin K Fajardo K Screening for asymptomatic bacteriuria in adults: evidence for the U.S. Preventive Services Task Force reaffirmation recommendation statement Ann Intern Med 2008 149 W20 W24 18591632 10.7326/0003-4819-149-1-200807010-00009-w1

[B7] National Collaborating Centre for Women’s and Children’s Health NICE clinical guideline 62; Antenatal care: routine care for the healthy pregnant woman 2008,

[B8] Schnarr J Smaill F Asymptomatic bacteriuria and symptomatic urinary tract infections in pregnancy Eur J Clin Invest 2008 38 Suppl 2 50 57 18826482 10.1111/j.1365-2362.2008.02009.x

[B9] Kenyon S Pike K Jones DR Brocklehurst P Marlow N Salt A Childhood outcomes after prescription of antibiotics to pregnant women with spontaneous preterm labour: 7-year follow-up of the ORACLE II trial Lancet 2008 372 1319 1327 10.1016/S0140-6736(08)61203-9 18804276

[B10] Bedford Russell AR Murch SH Could peripartum antibiotics have delayed health consequences for the infant? BJOG 2006 113 758 765 10.1111/j.1471-0528.2006.00952.x 16827757

[B11] Ashkenazi-Hoffnung L Melamed N Ben-Haroush A Livni G Amir J Bilavsky E The association of intrapartum antibiotic exposure with the incidence and antibiotic resistance of infantile late-onset serious bacterial infections Clin Pediatr (Phila) 2011 50 827 833 10.1177/0009922811406260 21885435

[B12] Widmer M Gulmezoglu AM Mignini L Roganti A Duration of treatment for asymptomatic bacteriuria during pregnancy Cochrane Database Syst Rev 2011 12 CD000491 22161364 10.1002/14651858.CD000491.pub2

[B13] Dashe JS Gilstrap LC Antibiotic use in pregnancy Obstet Gynecol Clin North Am 1997 24 617 629 10.1016/S0889-8545(05)70326-0 9266582

[B14] Reeves DS A perspective on the safety of antibacterials used to treat urinary tract infections J Antimicrob Chemother 1994 33 Suppl A 111 120 7928828 10.1093/jac/33.suppl_a.111

[B15] RIVM, SWAB Nethmap 2010: Consumption of antimicrobial agents and antimicrobial resistance among medically important bacteria in the Netherlands 2010,

[B16] Nederlandse Vereniging voor Obstetrie en Gynaecologie NVOG Guideline: urineweginfectie in de zwangerschap 2011,

[B17] Van Haaren KAM Visser HS Van Vliet S Timmermans AE Yadava R Geerlings SE Ter Riet G Van Pinxteren B NHG-Standaard Urineweginfectie (Tweede herziening) Huisarts Wet 2005 8 341 352

[B18] Stenqvist K Dahlen-Nilsson I Lidin-Janson G Lincoln K Oden A Rignell S Bacteriuria in pregnancy. Frequency and risk of acquisition Am J Epidemiol 1989 129 372 379 2912046 10.1093/oxfordjournals.aje.a115140

[B19] Smaill F Vazquez JC Antibiotics for asymptomatic bacteriuria in pregnancy Cochrane Database Syst Rev 2007 2 CD000490 17443502 10.1002/14651858.CD000490.pub2

[B20] Sheffield JS Cunningham FG Urinary tract infection in women Obstet Gynecol 2005 106 1085 1092 10.1097/01.AOG.0000185257.52328.a2 16260529

[B21] Mignini L Carroli G Abalos E Widmer M Amigot S Nardin JM Accuracy of diagnostic tests to detect asymptomatic bacteriuria during pregnancy Obstet Gynecol 2009 113 346 352 19155905 10.1097/AOG.0b013e318194f109

[B22] Schlager TA Smith DE Donowitz LG Perineal cleansing does not reduce contamination of urine samples from pregnant adolescents Pediatr Infect Dis J 1995 14 909 911 10.1097/00006454-199510000-00020 8584324

[B23] Anderson BL Simhan HN Simons KM Wiesenfeld HC Untreated asymptomatic group B streptococcal bacteriuria early in pregnancy and chorioamnionitis at delivery Am J Obstet Gynecol 2007 196 524 525 17547879 10.1016/j.ajog.2007.01.006

[B24] Whalley P Bacteriuria of pregnancy Am J Obstet Gynecol 1967 97 723 738 5335276 10.1016/0002-9378(67)90458-9

[B25] van Os MA van der Ven JA Kleinrouweler CE Pajkrt E De ME Van WA Preventing preterm birth with progesterone: costs and effects of screening low risk women with a singleton pregnancy for short cervical length, the Triple P study BMC Pregnancy Childbirth 2011 11 77 10.1186/1471-2393-11-77 22023876 PMC3214137

